# Cytotoxic Activity of Essential Oils from Middle Eastern Medicinal Plants on Malignant Keratinocytes

**DOI:** 10.3390/molecules30132844

**Published:** 2025-07-03

**Authors:** Rima Othman, Vanessa Moarbes, Muriel Tahtouh Zaatar, Diane Antonios, Rabih Roufayel, Marc Beyrouthy, Ziad Fajloun, Jean-Marc Sabatier, Marc Karam

**Affiliations:** 1 Department of Pulmonary Medicine and Critical Care, Johns Hopkins University, Baltimore, MD 21205, USA; remaothman3@gmail.com; 2 Department of Biology, Faculty of Sciences, University of Balamand, Al-Kourah, Tripoli P.O. Box 100, Lebanon; vanessa.moarbes@mail.mcgill.ca (V.M.); marckaram1@gmail.com (M.K.); 3 Department of Biological and Physical Sciences, American University in Dubai, Dubai P.O. Box 28282, United Arab Emirates; 4 Laboratory of Toxicology, Faculty of Pharmacy, Saint Joseph University of Beirut, Damascus Road, Beirut P.O. Box 11-5076, Lebanon; diane.antonios@usj.edu.lb; 5 College of Engineering and Technology, American University of the Middle East, Egaila 54200, Kuwait; rabih.roufayel@aum.edu.kw; 6 Department of Agriculture and Food Engineering, School of Engineering, Holy Spirit University of Kaslik, Jounieh P.O. Box 446, Lebanon; marcelbeyrouthy@usek.edu.lb; 7 Department of Cell Culture, Laboratory of Applied Biotechnology (LBA3B), Azm Center for Research in Biotechnology and Its Applications, EDST, Lebanese University, Tripoli 1300, Lebanon; 8 Faculty of Sciences 3, Lebanese University, Michel Slayman Tripoli Campus, Ras Maska 1352, Lebanon; 9 Inst Neurophysiopathol (INP), CNRS, Aix-Marseille Université, 13385 Marseille, France

**Keywords:** anti-cancer effect, MTT assay, essential oil, HaCat, A5 cell lines, II4 cell lines, *Juniperus excelsa* M. Bieb. (Cupressaceae), *Lavandula vera* DC. (Lamiaceae), *Salvia fruticosa Mill.* (Lamiaceae)

## Abstract

Skin cancer, including melanoma and non-melanoma cancers (basal and squamous cell carcinomas), is the most common type of cancer. UV radiation, family history, and genetic predisposition are the main risk factors. Although surgical excision is the standard treatment, essential oils are attracting growing interest for their anti-cancer effects. This study tested the effects of *Juniperus excelsa* M. Bieb. (Cupressaceae), *Lavandula vera* DC. (Lamiaceae), and *Salvia fruticosa (Mill)*. (Lamiaceae) essential oils extracted from Middle Eastern medicinal plants on HaCaT (normal), A5 (benign), and II4 (low-grade malignant) keratinocytes. Essential oils were extracted from *Juniperus excelsa*, *Lavandula vera*, and *Salvia libanotica* using steam distillation and then were chemically analyzed. The oils were sterilized, dissolved in DMSO, and prepared at concentrations of 0.75, 0.5, and 0.25 mg/mL. Human keratinocyte (HaCaT), benign (A5), and malignant (II4) cell lines were cultured in DMEM and treated with the essential oils for 24 or 48 h. Cell viability was assessed using the Trypan Blue Exclusion Test, while cell proliferation was evaluated using the MTT assay. Statistical analysis was performed using ANOVA with appropriate post hoc tests, considering *p* < 0.05 as significant. The results show that *J. excelsa* is cytotoxic but lacks selectivity, limiting its efficacy. In contrast, *L. vera* and *S. fruticosa* preferentially target malignant cells, particularly at low concentrations, while sparing normal cells. These oils have dose-dependent anticancer effects, with *L. vera* efficacy increasing as the concentration increases. In conclusion, *L. vera* and *S. fruticosa* are promising candidates for the treatment of skin cancer, although further in vivo studies are required.

## 1. Introduction

Skin cancer is the most prevalent form of cancer globally, representing a diverse and complex group of malignancies that arise from various cellular components within the skin. These cancers can affect individuals of all ethnic backgrounds and age groups, underscoring their widespread impact on public health. Among these, cutaneous carcinomas—or skin cancers—are broadly categorized into two primary groups: melanoma and non-melanoma skin cancers (NMSCs). The latter includes the two most common types, basal cell carcinoma (BCC) and squamous cell carcinoma (SCC), which together account for the majority of skin cancer diagnoses worldwide [1].

Skin cancer is the most commonly diagnosed cancer globally and encompasses a range of malignancies, including melanoma and non-melanoma skin cancers (NMSCs) such as basal cell carcinoma (BCC) and squamous cell carcinoma (SCC) [2]. Ultraviolet (UV) radiation is the leading environmental risk factor, while genetic predisposition also plays a significant role [3]. The skin’s structure, particularly the keratinocyte-rich epidermis, is critical to understanding cancer initiation. Keratinocytes mature as they migrate through the epidermal layers and are vulnerable to UV-induced DNA damage, which may lead to tumorigenesis [4,5].

Although surgical excision remains the cornerstone of treatment for most forms of skin cancer, particularly when diagnosed early, there is a growing need for complementary therapies that can reduce recurrence, improve outcomes, and lower treatment-associated morbidity. In recent years, natural compounds derived from plants—especially phytochemicals found in essential oils and extracts—have emerged as promising candidates for cancer prevention and therapy. These bioactive molecules have long been used in traditional medicine to promote skin health and regeneration, and recent studies suggest that they may also possess potent anticancer properties [6].

Our research focuses on exploring the anticancer potential of specific native Middle Eastern plants, including *Juniperus excelsa* M. Bieb. (Cupressaceae) (juniper berries), *Lavandula vera* DC. (Lamiaceae) (lavender flowers), and *Salvia fruticosa* (Mill.). (Lamiaceae) (Lebanese sage). We investigate the cytotoxic effects of their extracts on keratinocytes, aiming to assess their viability as novel therapeutic agents against skin cancer. By combining ethnobotanical knowledge with modern biomedical research, this study seeks to contribute to the growing field of plant-based anticancer treatments, with a particular emphasis on their application in dermatological oncology [7,8].

Essential oils, which are concentrated volatile compounds extracted from aromatic plants, have long been valued in traditional medicine for their antimicrobial, anti-inflammatory, and regenerative properties. In recent decades, research has increasingly focused on their potential anticancer activities, particularly through mechanisms relevant to skin cancer pathology. The bioactive constituents of essential oils, such as terpenes, phenolics, flavonoids, and aldehydes, can interact with multiple cellular pathways involved in carcinogenesis, including those governing oxidative stress, inflammation, apoptosis, and cell cycle regulation [9].

This study primarily focused on evaluating the cytotoxic potential of selected essential oils against keratinocyte cell lines, including benign and malignant variants, without delving into the underlying mechanisms of cell death. By employing two fundamental assays—the Trypan Blue Exclusion Test and the MTT assay—we assessed whether the tested essential oils could induce cell death and quantified the percentage of dead cells relative to the DMSO control. While the mechanistic pathways remain to be elucidated, this work provides preliminary evidence of cytotoxic activity, forming a basis for future mechanistic investigations.

## 2. Results

### 2.1. Chemical Composition of Essential Oils

The chemical composition of the essential oils (EOs) was first determined using Gas Chromatography–Mass Spectrometry (GC-MS), Co-injection Gas Chromatography (Co-GC), and Retention Index (Ri) comparisons. The main constituents identified in each oil are presented in Table 1, along with their extraction yields and relative percentages.

Juniperus excelsa oil was predominantly composed of α-pinene (86.8%), while Salvia libanotica was rich in 1,8-cineole (48.7%) and β-caryophyllene (30.8%). The main components of Lavandula vera oil were linalool (42.5%) and terpinen-4-ol (10.5%). These results align with previous studies [8,10,11] that have identified similar major constituents in these species, although minor variations may result from differences in geographical origin, seasonal timing of harvest, or extraction techniques.

A more detailed presentation of the identified compounds, including their potential biological relevance, is essential as these chemical constituents likely contribute to the observed cytotoxic effects of the oils. For example, α-pinene has known anti-proliferative and pro-apoptotic activities, 1,8-cineole exhibits anti-inflammatory and anticancer properties, and linalool is widely reported for its cytotoxic potential against various cancer cell lines.

Following the chemical characterization, the essential oils were subjected to in vitro cytotoxicity assays to assess their effects on the HaCaT, A5, and II4 cell lines.

### 2.2. Assessment of Essential Oils’ Effect on Cell Viability

#### 2.2.1. Effect of *Juniperus excelsa* M. Bieb. (Cupressaceae)

The potential dose-dependent effect of *J. excelsa* essential oil was assessed in each of the cell lines of interest in which the essential oil, in its three studied concentrations, was revealed to significantly promote a dose-independent cell death. Indeed, increasing the concentration of this essential oil from 0.25 mg/mL to 0.5 mg/mL and 0.75 mg/mL induced a roughly constant high cell death percentage. These cell death percentages were demonstrated to comprise an elevated extent of statistical significance upon comparison with the control representative cells of each stage treated with DMSO alone, where the % cell death displayed minimal irrelevant percentages of 8.83% in HaCaT, 4.25% in A5, and 10.07% in II4.

**Figure 1:**
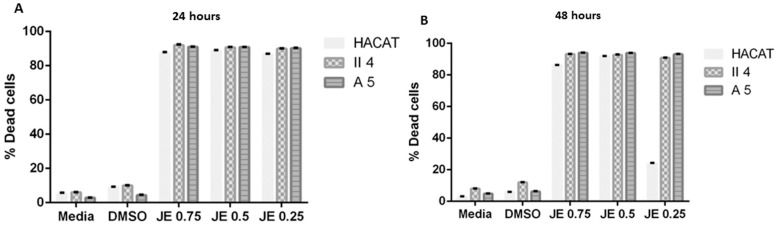
Comparison of *Juniperus excelsa* essential oil’s effect on cell viability among keratinocytes representative of different skin cancer stages following 24 and 48 h treatments shown in (**A**,**B**) respectively. Data are presented as mean ± SEM of three experiments, performed in triplicate. Essential oil treatments of tumorigenic A5 and II4 keratinocytes were compared in reference to essential oil treatments of control non-tumorigenic HaCaT keratinocytes. Significance was detected at (*p* < 0.5).

In addition to being shown to be dose independent, *J. excelsa* essential oil’s effect on HaCaT, A5, and II4 keratinocyte cell viability was found to be time independent whereby prolonging the treatment exposure period from 24 h to 48 h did not significantly alter the cytotoxic effect against any cell line scrutinized (Figure 1).

Furthermore, *J. excelsa* essential oil’s effect on cell viability was shown throughout our investigation to be non-differential since the % cell death induction of each of the tested concentrations, 0.25 mg/mL, 0.5 mg/mL and 0.75 mg/mL, in benign A5 and low-grade malignant II4 keratinocytes demonstrated statistical insignificance when compared to the HaCaT control cell line’s % cell death; this indiscriminate effect and insignificant % cell death variation were further conserved upon treatment exposure prolongation to 48 h without any further significant amendment (Figure 1).

#### 2.2.2. Effect of *Lavandula vera* DC. (Lamiaceae)

The *L. vera* essential oil’s dose-associated effect was assessed in each of the previously described target cell lines in which the three investigated essential oil concentrations were capable of significantly instigating a concentration-dependent % cell death. Moreover, except for the effect displayed by the *L. vera* essential oil concentration of 0.25 mg/mL on HaCaT keratinocyte cell death, whose significance was only moderately high, the previously mentioned essential oil-attributed cell death percentages contained a high degree of statistical significance upon comparison with the control representative cells of each stage treated with DMSO alone.

**Figure 2:**
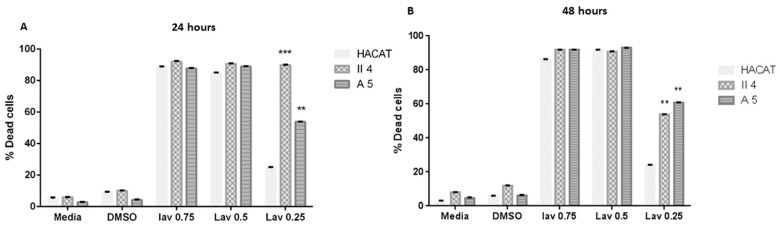
Comparison of *Lavendula vera* essential oil’s effect on cell viability among keratinocytes representative of different skin cancer stages following 24 and 48 h treatments shown in (**A**,**B**) respectively. Data are presented as mean ± SEM of three experiments, performed in triplicate. Essential oil treatments of tumorigenic A5 and II4 keratinocytes were compared in reference to essential oil treatments of control non-tumorigenic HaCaT keratinocytes. ** *p* < 0.01; *** *p* < 0.001 compared to control. Significance was detected at (*p* < 0.5).

Despite being shown to be dose dependent, *L. vera* essential oil’s effect on HaCaT, A5, and II4 keratinocyte cell viability was found to have time-independent features whereby prolonging the treatment exposure period from 24 h to 48 h failed to induce any significant alterations in the cytotoxic effects against any inspected cell line (Figure 2).

Of particular interest, *L. vera* essential oil’s cytotoxic effect at low concentrations strikingly manifested itself throughout our investigation as highly discriminatory between the investigated cell lines with a preferential toxicity towards cancerous keratinocytes in which the low concentrations, which were shown to be non-toxic to normal cells, exhibited lethal effects on the human skin cancer cells examined. Indeed, 0.25 mg/mL *L. vera* essential oil induced a relatively slight cell death percentage of 24.98% in control non-tumorigenic HaCaT keratinocytes, in contrast to comparatively preeminent cell death percentages of 53.85% cell death in benign A5 keratinocytes and 89.93% cell death in low-grade malignant II4 keratinocytes (Figure 2). Accordingly, at this essential oil concentration, *L. vera*’s formerly stated cytotoxic effect on A5 and II4 keratinocytes was revealed to encompass a prominent grade of statistical significance upon comparison with the % cell death attributed to the HaCaT control cell line. This significance in the discriminant and preferential cell death effect was preserved without any further noteworthy adjustments upon treatment exposure prolongation to 48 h (Figure 2).

#### 2.2.3. Effect of *Salvia fruticosa* (Mill.) (Lamiaceae)

*S. fruticosa* essential oil’s tentative dose-reliant effect was evaluated in each of the previously demarcated control and tumorigenic target cell lines in which the three explored essential oil concentrations were proficient at significantly prompting a concentration contingent cell death. Moreover, excluding the effect presented by the *S. fruticosa* 0.25 mg/mL essential oil concentration on HaCaT keratinocyte cell death, whose significance was only temperately elevated, this essential oil-induced cell death percentages were shown to have an eminent degree of statistical significance when compared to the archetypal control cells of each stage subjected to DMSO treatment.

**Figure 3:**
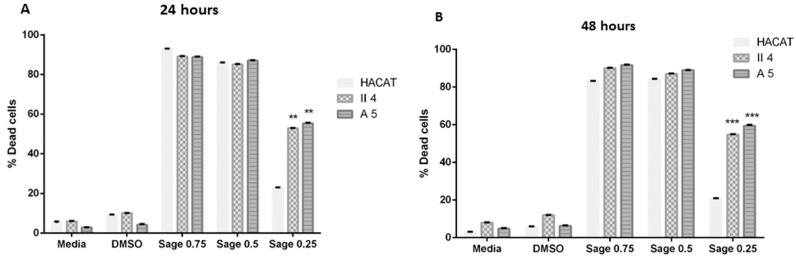
Comparison of *Salvia fruticosa* essential oil’s effect on cell viability among keratinocytes representative of different skin cancer stages following 24 and 48 h treatments shown in (**A**,**B**) respectively. Data are presented as mean ± SEM of three experiments, performed in triplicate. Essential oil treatments of tumorigenic A5 and II4 keratinocytes were compared in reference to essential oil treatments of control non-tumorigenic HaCaT keratinocytes. ** *p* < 0.01; *** *p* < 0.001 compared to control. Significance was detected at (*p* < 0.5).

Notwithstanding the former dose-dependent cell death influence, *S. fruticosa* essential oil’s effect on HaCaT, A5, and II4 keratinocyte cell viability was revealed to have time-independent characteristics wherein lengthening the treatment exposure period from 24 h to 48 h proved unsuccessful at causing any significant shifts in the studied cytotoxic effects against any inspected cell line (Figure 3).

Of particular interest, *S. fruticosa* essential oil’s cytotoxic effect at low concentrations strikingly established itself throughout the course of our inquiry as possessing highly discriminant features between the considered cell lines, with toxicity favoring cancerous keratinocytes in which low concentrations, which were found to be non-toxic to normal cells, exhibited lethal effects on the human skin cancer cells examined. Indeed, 0.25 mg/mL *S. fruticosa* essential oil prompted a relatively trivial cell death percentage of 22.88% in non-tumorigenic HaCaT keratinocytes in contrast to a reasonably dominant cell death in benign A5 keratinocytes of 55.39% and in low-grade malignant II4 keratinocytes of 52.89%. Consequently, at this essential oil concentration, *S. fruticosa*’s previously quantified cytotoxic effect on A5 and II4 keratinocytes exposed a conspicuous grade of statistical significance upon comparison with the % cell death ascribed to the HaCaT control cell line. This significance in the discriminant and privileged cell death effect was devoid of any further remarkable variations upon treatment exposure continuation to 48 h (Figure 3).

### 2.3. Evaluation of Essential Oils’ Effect on Cell Proliferation

#### 2.3.1. Effect of *Juniperus excelsa* M. Bieb. (Cupressaceae)

**Figure 4:**
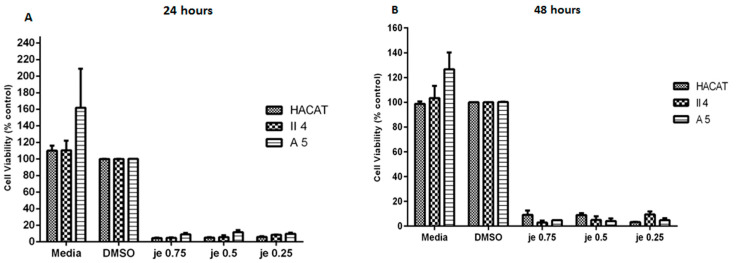
Comparison of *Juniperus excelsa* essential oil’s effect on metabolic activity (MTT assay), used as an indicator of cell viability among keratinocytes representative of the different skin cancer stages following 24 and 48 h treatments shown in (**A**,**B**) respectively. Data are presented as mean ± SEM of three experiments, performed in triplicate. Essential oil treatments of tumorigenic A5 and II4 keratinocytes were compared in reference to essential oil treatments of control non-tumorigenic HaCaT keratinocytes. Control represents DMSO-treated cells. Significance was detected at (*p* < 0.5).

Following the described MTT assay, the probable dose-dependent effect of *J. excelsa* essential oil on cell proliferation was quantified and found to have prominent inhibitory effects on HaCaT, A5, and II4 keratinocyte cell proliferation. Indeed, increasing the concentration of this essential oil from 0.25 mg/mL to 0.5 mg/mL and 0.75 mg/mL induced an approximately constant growth suppression displayed as a notable decrease in cell viability: 5.98%, 5.16%, and 4.57%, respectively (Figure 4). These cell viability percentages were demonstrated to comprise an elevated extent of statistical significance upon comparison with the control representative cells of each stage treated with DMSO alone.

In addition to being shown to be dose independent, *J. excelsa* essential oil’s effect on HaCaT, A5, and II4 keratinocyte cell proliferation displayed itself as time independent whereby prolonging the treatment exposure period from 24 h to 48 h did not significantly alter the growth inhibitory effects against any cell line scrutinized.

Furthermore, *J. excelsa* essential oil’s effect on cell proliferation was shown throughout our investigation to be non-differential since the percentage cell viability of each of the tested concentrations, 0.25 mg/mL, 0.5 mg/mL, and 0.75 mg/mL, in benign A5 and low-grade malignant II4 keratinocytes demonstrated statistical insignificance when compared to the HaCaT control cell line’s percentage cell viability; this indiscriminate effect and insignificant percentage cell viability variation were further conserved upon treatment exposure prolongation to 48 h without any further significant amendment (Figure 4).

#### 2.3.2. Effect of *Lavandula vera* DC. (Lamiaceae)

**Figure 5:**
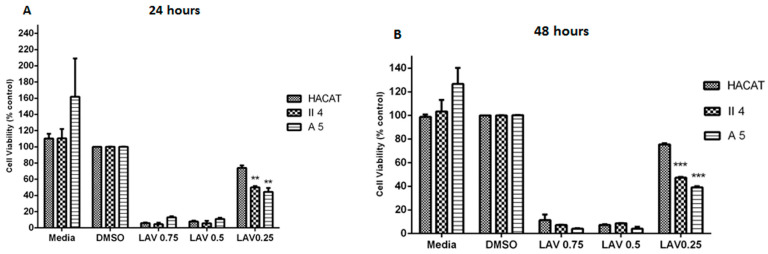
Comparison of *Lavendula vera* essential oil’s effect on metabolic activity (MTT assay), used as an indicator of cell viability among keratinocytes representative of different skin cancer stages following 24 and 48 h treatments as shown in (**A**,**B**) respectively. Data are presented as mean ± SEM of three experiments, performed in triplicate. Essential oil treatments of tumorigenic A5 and II4 keratinocytes were compared in reference to essential oil treatments of control non-tumorigenic HaCaT keratinocytes. Control represents DMSO-treated cells. ** *p* < 0.01; *** *p* < 0.001 compared to control. Significance was detected at (*p* < 0.5).

After performing the MTT assay, the dose-dependent effect of *L. vera* essential oil on cell proliferation was calculated in the previously described target cell lines; the three investigated essential oil concentrations were capable of significantly influencing the attributed cell proliferation in a concentration-dependent manner as evidenced from the fluctuations in percentage cell viability. Moreover, except for the effect displayed by the *L. vera* essential oil concentration of 0.25 mg/mL on HaCaT keratinocyte cell proliferation, whose significance was only moderately high, the essential oil-attributed cell viability percentages contained a high degree of statistical significance upon comparison with the control representative cells of each stage treated with DMSO alone in which the % cell viability was revealed as 100% due to its minimal irrelevant growth suppression in the three cell line of interest (Figure 5).

Despite being shown to be dose dependent, the *L. vera* essential oil effect on HaCaT, A5, and II4 keratinocyte cell viability exhibited time-independent features wherein prolonging the treatment exposure period from 24 h to 48 h failed to induce any significant alterations in the growth suppression effects against any inspected cell line (Figure 5).

Of particular interest, at low concentrations, *L. vera* essential oil’s effect on cell proliferation strikingly manifested itself throughout our investigation as highly discriminant between the investigated cell lines, with a preferential growth inhibitory trend towards cancerous keratinocytes in which the low concentrations, which were shown to be ineffective in normal cells, exhibited potent growth inhibitory effects on the human skin cancer cells examined. Indeed, 0.25 mg/mL *L. vera* essential oil induced a relatively slight decline in percentage cell viability of 73.94% in control non-tumorigenic HaCaT keratinocytes, in contrast to a comparatively preeminent cell viability reduction in benign A5 keratinocytes of 44.31% and in low-grade malignant II4 keratinocytes of 49.91% (Figure 5). Accordingly, at this essential oil concentration, *L. vera*’s formerly stated growth inhibitory effect on A5 and II4 keratinocytes was revealed to encompass a prominent grade of statistical significance upon comparison with the % cell viability attributed to the HaCaT control cell line This significance in the discriminant and preferential cell death effect was preserved without any further noteworthy adjustments upon treatment exposure prolongation to 48 h (Figure 5).

#### 2.3.3. Effect of *Salvia fruticosa* Mill. (Lamiaceae)

**Figure 6:**
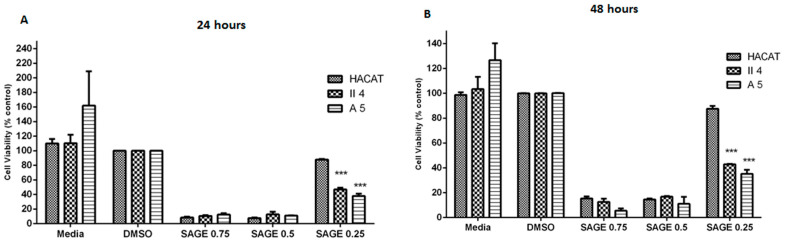
Comparison of *Salvia fruticosa* essential oil effect on metabolic activity (MTT assay), used as an indicator of cell viability among keratinocytes representative of different skin cancer stages following 24 and 48 h treatments as shown in (**A**,**B**) respectively. Data are presented as mean ± SEM of three experiments, performed in triplicate. Essential oil treatments of tumorigenic A5 and II4 keratinocytes were compared in reference to essential oil treatments of control non-tumorigenic HaCaT keratinocytes. *** *p* < 0.001 compared to control. Significance was detected at (*p* < 0.5).

Following the MTT assay, the prospective dose-dependent effect of *S. fruticosa* essential oil on cell proliferation was measured in the target cell lines in which the three considered essential oil concentrations were proficient at significantly influencing the attributed cell proliferations in a concentration-dependent fashion as evidenced by apparent fluctuations in the percentage cell viability. For instance, upon increasing the essential oil concentration, significant percentage cell viability reductions were observed. Furthermore, excluding the effect exhibited by the *S. fruticosa* 0.25 mg/mL essential oil concentration on HaCaT keratinocyte cell proliferation, whose significance was only moderately high, this essential oil’s associated cell viability percentages were confirmed to have an elevated grade of statistical significance upon comparison with the control representative cells of each stage treated with DMSO alone, where the % cell viability was considered as 100% due to its minimal irrelevant growth suppression effects in the three cell lines of interest (Figure 6).

Although established as dose dependent, the *S. fruticosa* essential oil effect on HaCaT, A5, and II4 keratinocyte cell proliferation exhibited time-independent features whereby prolonging the treatment exposure period from 24 h to 48 h failed to induce any significant alterations in the growth suppression effects against any examined cell line (Figure 6).

Of particular importance, at low essential oil concentrations, *S. fruticosa* oil’s effect on cell proliferation strikingly expressed itself throughout our investigation as highly discriminant between the probed cell lines with a preferential growth inhibitory trend towards cancerous keratinocytes, wherein low concentrations, which were shown to be ineffective in normal cells, exhibited potent growth inhibitory effects on the human skin cancer cells examined. Indeed, 0.25 mg/mL *S. fruticosa* essential oil induced a relatively slight proliferation decline in the control non-tumorigenic HaCaT keratinocytes whose percentage cell viability was 87.90%, in contrast to a comparatively preeminent cell proliferation reduction in benign A5 keratinocytes whose percentage cell viability was 37.86% and in low-grade malignant II4 keratinocytes whose percentage cell viability was 46.91% (Figure 6). Accordingly, at this essential oil concentration, *S. fruticosa*’s growth inhibitory effects on A5 and II4 keratinocytes were revealed to encompass a prominent grade of statistical significance upon comparison with the % cell viability attributed to the HaCaT control cell line. This significance in the discriminant and preferential cell death effect was preserved without any further noteworthy adjustments upon treatment exposure prolongation to 48 h (Figure 6).

## 3. Materials and Methods

### 3.1. Chemicals and Reagents

Penicillin Streptomycin, L-Glutamine, HEPES(4-(2-hydroxyethyl)-1-piperazineethanesulfonic acid), heat-inactivated fetal bovine serum (FBS), Phosphate Buffer Saline (PBS), Dimethyl Sulfoxide (DMSO), Trypsin EDTA, and Trypan Blue Exclusion Dye were purchased from Sigma-Aldrich Co. (Burlington, MA, USA); Dulbecco Modified Eagle Media (DMEM) was obtained from Gibco Life Technologies (Carlsbad, CA, USA); the Vybrant MTT 3-(4,5-dimethylthiazol-2,5-diphenyltetrazoliumbromide) Cell Proliferation Assay Kit V-13154 was obtained from Molecular Probes subdivision of Thermo Fisher Scientific. 168 Third Avenue. Waltham, MA, USA.

### 3.2. Plant Collection and Essential Oil Isolation and Analysis

Essential oils from *Juniperus excelsa* M. Bieb. (Cupressaceae) berries, *Lavandula vera* DC. (Lamiaceae) flowers, and *Salvia fruticose* Mill. (Lamiaceae) leaves collected from Kartaba and Nahr Ibrahim during the months of October (*J. excelsa*) and May (*L. vera* and *S. fruticosa*) 2011 were kindly supplied by Dr. Marc Beyrouthy (USEK University). Aerial plant fragments were immediately pulverized and subjected to steam distillation for 3 h using a Clevenger type apparatus for the isolation of their essential oils. Then, the obtained essential oils were collected, dried over anhydrous sodium sulfate, filtrated, and stored at 4 °C in tightly closed, dark glass vials wrapped in aluminum foil to prevent oxygen and light exposure for a maximum period of one week until submission to Mass Spectrometry (MS), Co-injection Gas Chromatography (CoGC), and Linear Retention Index (Ri) analyses for the detection of their major constituents.

The botanical identity of the plant materials was confirmed by Dr. Marc Beyrouthy (USEK University), using regional floristic references. However, no voucher specimens were deposited in a public herbarium. The plant materials were collected and authenticated based on morphological features, and representative samples are archived in the private botanical collection of Dr. Beyrouthy for future reference.

### 3.3. Essential Oil Dissolution

In order to facilitate the dispersion of the oils in the aqueous culture medium prior to each employment, essential oils, sterilized with 0.45 µm Millipore filters, were dissolved in 100% DMSO to prepare 10% (*v*/*v*) stock solutions. Working concentrations of 0.75, 0.5, and 0.25 mg/mL were freshly prepared by direct dilution into complete culture medium, maintaining a final DMSO concentration of ≤0.1% (*v*/*v*) in all treated wells. These concentrations were further screened with the purpose of eluting three optimal concentrations (0.75 mg/mL, 0.5 mg/mL, and 0.25 mg/mL) to be employed in the targeted investigations.

#### 3.3.1. GC Analyses

Analytical gas chromatography was performed on a Thermo Electron Corporation (subdivision of Thermo Fisher Scientific. 168 Third Avenue. Waltham, MA, USA) gas chromatograph fitted with a flame ionization detector (FID), a DB-5 MS capillary column (30 m × 0.25 mm) with 0.1 μm film thickness, or a fused silica HP Innowax polyethylene glycol capillary column (50 m × 0.20 mm, film thickness 0.20 μm). Helium was the carrier gas (0.7 mL/min). The column temperature was initially set to 35 °C before being gradually increased to 85 °C at 5 °C/min, held for 20 min at 85 °C, raised to 300 °C at 10 °C/min, and finally held for 5 min at 300 °C. Diluted 1 μL samples (1/100, *v*/*v*) were injected at 250 °C manually and in the splitless mode. Flame ionization detection (FID) was performed at 310 °C.

#### 3.3.2. GC–MS Analyses

The GC/MS analyses were performed using an Agilent 6890 gas chromatograph coupled with a 5975 Mass Detector (by Agilent 5301 Stevens Creek Blvd Santa Clara, CA, USA). The 7683 B auto sampler injected 1 μL of each diluted oil sample (1/100, *v*/*v*). A fused silica capillary column DB-5 MS (30 m × 0.25 mm internal diameter, film thickener 0.1 μm) or a fused silica HP Innowax polyethylene glycol capillary column (50 m × 0.20 mm, film thickness 0.20 μm) was used. Helium was the carrier gas (0.7 mL/min). The oven temperature program was identical to that described above (cf. GC Analysis). The mass spectra were recorded at 70 eV with an ion source temperature of 310 °C and a transfer line heated to 320 °C. The acquisition was recorded in full scan mode (50–400 *m*/*z*).

#### 3.3.3. Qualitative and Quantitative Analysis

Most constituents were identified by gas chromatography by comparing their retention indices (RIs) with those from the literature [12,13] or with those of authentic compounds obtained from Sigma-Aldrich (Lebanon and France). The retention indices were determined relative to a homologous series of n-alkanes (C_8_ to C_24_) analyzed under the same operating conditions. Concomitantly, their mass spectra on both columns were compared with those provided in the NIST and Wiley 275 libraries, our home-made library constructed with pure compounds and EOs of known composition, or with mass spectra from the literature [14,15]. The relative concentration of each component was calculated based on the GC peak areas without using correction factors.

### 3.4. Cell Culture

The formerly described spontaneously immortalized non-tumorigenic Human Keratinocyte (HaCaT) cell line and its benign (A5) and malignant (II4) clones were kindly provided by Dr. Marwan Sabban (American University of Beirut). The provided cells were cultured at normal 2 mM Calcium concentrations in Dulbecco’s modified Eagle’s medium (DMEM) supplemented with 10% heat-inactivated fetal bovine serum (FBS), 100 μg/mL penicillin–streptomycin, L-Glutamine (final concentration: 2 mM), and 1% HEPES and maintained in a humidified incubator containing 95% air and 5% CO_2_ at 370 °C. Throughout the investigation course, the cell lines had their medium renewed and replenished every 2 days and were passaged upon attaining 70% confluence (every 7 to 10 days) via Trypsin–EDTA treatment. The passage methodology consisted of washing the attached cells with 5 mL (0.05%) of EDTA in order to remove the serum, Ca^2+^, Mg^2+^, and all factors that inhibit trypsin, followed by incubation with 5 mL (0.05%) of EDTA for a maximum of 6 min in order to accomplish desmosome destruction displayed as microscopically visible wide intercellular spaces. Once this was achieved, the cells were then incubated with 1:1 Trypsin (0.025%)–EDTA (0.05%) for up to 3 min until the cells came off the plastic upon shaking. Subsequent to cell detachment, an equal volume of fresh medium was added to the culture flask to inactivate trypsin, and the cells were then suspended, transferred into falcon tubes, and centrifuged at 900× *g* for 5 min at 40 °C. Finally, the resulting cell pellet was re-suspended in fresh medium and split into a maximal ratio of 1:5 into cell culture flasks for future seeding, treatment, and analysis.

### 3.5. Cell Viability Analysis: Trypan Blue Exclusion Test

Prior to the viability assays, HaCaT, A5, and II4 cells were plated in 24-well plates at a density of 1 × 10^6^ cells/well in 1 mL of medium to ensure consistent conditions. After seeding, the cells were incubated for 24 h at 37 °C in 5% CO_2_ to allow attachment. The medium was then replaced with fresh medium containing essential oils of *J. excelsa*, *L. vera*, or *S. fruticose Mil* at final concentrations of 0.75, 0.5, or 0.25 mg/mL. Treatments were performed in triplicate alongside a negative control (0.01% DMSO) and a positive control (culture only). Cells were incubated for an additional 24 or 48 h under the same conditions. Cytotoxicity was assessed using the Trypan Blue Exclusion Test: cells were harvested, mixed with an equal volume of Trypan Blue, and counted using a hemocytometer. Percent viability (unstained cells/total cells × 100) and percent cell death (stained cells/total cells × 100) were calculated accordingly.

### 3.6. Cell Proliferation Examination: MTT Assay

The MTT assay measures mitochondrial metabolic activity, which is proportional to the number of metabolically active cells and is commonly used as an indicator of cell proliferation. For proliferation analysis, HaCaT, A5, and II4 cells were seeded in 96-well plates at 1000 cells/well in 200 μL of medium and then incubated for 24 h at 37 °C with 5% CO_2_ to allow cell attachment. The medium was then replaced with fresh medium containing *J. excelsa*, *L. vera*, or *S. fruticosa* essential oils at final concentrations of 0.75, 0.5, or 0.25 mg/mL. All treatments were performed in triplicate alongside a negative control (0.01% DMSO) and a positive control (media only). After 24 and 48 h of incubation, cell proliferation was assessed using the MTT assay [16], which measures metabolic activity via conversion of yellow tetrazolium to blue formazan. The assay was conducted with the Vybrant MTT kit. Briefly, the medium was replaced with 100 μL of fresh medium, and 10 μL of 12 mM MTT stock solution was added, followed by a 4 h incubation at 37 °C. A test blank (MTT without cells) was included. After incubation, 85 μL of medium was removed and replaced with 50 μL of DMSO to solubilize the formazan crystals, followed by a 10 min incubation. The absorbance was read at 540 nm, and the % viability was calculated as: (mean absorbance of treated wells/mean absorbance of untreated control) × 100.

### 3.7. Statistical Data Analysis

All triplicate experimental values obtained are presented as the mean ± standard error mean (SEM). The experimental data were analyzed using Graph Pad Prism 5 statistical software (GraphPad Software Inc., San Diego, CA, USA), and the statistical significance was assessed via one-way Anova Tukey test for single step multiple comparisons, one-way Anova Dunnet test for comparison with control, and two-way Anova Bonferroni test for comparisons among cell lines, with * *p* values < 0.05 considered as statistically significant.

## 4. Discussion

Skin cancer, similar to many malignancies, often results from a combination of chronic inflammation, oxidative DNA damage, and impaired regulation of cell proliferation. Exposure to ultraviolet (UV) radiation not only directly damages DNA in epidermal cells, particularly keratinocytes and melanocytes, but also generates reactive oxygen species (ROS) that trigger chronic oxidative stress and inflammation—two hallmarks of tumor initiation and progression. Furthermore, UV radiation can induce local immunosuppression, allowing mutated cells to evade immune surveillance and continue proliferating [17,18,19,20].

Essential oils offer a promising countermeasure to these pathological processes. Many have demonstrated potent antioxidant capabilities, such as neutralizing ROS and protecting skin cells from UV-induced damage. Others exhibit anti-inflammatory effects by modulating signaling pathways that are known to be upregulated in both melanoma and non-melanoma skin cancers. Several essential oils also promote apoptosis (programmed cell death) in malignant cells by activating intrinsic mitochondrial pathways or extrinsic death receptor pathways while leaving normal skin cells relatively unharmed—a feature that makes them attractive candidates for targeted cancer therapy [17,21].

Notably, plant-derived essential oils such as those from *Juniperus excelsa* M. Bieb. (Cupressaceae), *Lavandula vera* DC. (Lamiaceae), and *Salvia fruticosa* (Mill.) (Lamiaceae) have been historically used in Middle Eastern folk medicine for treating skin conditions, wounds, and infections. Recent in vitro studies suggest that extracts from these plants may also exert cytotoxic effects on human carcinoma cell lines, including keratinocytes transformed by UV exposure. For instance, components such as linalool, carvacrol, and α-pinene—found in these oils—have been shown to inhibit cell proliferation, induce apoptosis, and reduce tumor angiogenesis. Their dual role in enhancing skin barrier function and exerting anticancer activity makes them particularly suitable for therapeutic exploration in cutaneous carcinomas [9,22].

The comparative analysis of the three essential oils highlights a critical distinction in their therapeutic potential. While *J. excelsa* demonstrates potent anticancer activity, its non-selective cytotoxicity diminishes its suitability for clinical use (Figure 1 and Figure 6). In contrast, *L. vera* and *S. fruticosa* show promising selective toxicity at lower doses, preferentially targeting malignant cells while sparing normal keratinocytes (Figure 2, Figure 4 and Figure 5). This cancer-selective behavior is a key criterion for developing effective and safe alternative therapies, particularly in dermatologic oncology where preserving healthy tissue integrity is paramount.

Furthermore, the observed dose-dependent but time-independent nature of *L. vera* and *S. fruticosa* suggests that therapeutic efficacy may be optimized through concentration modulation rather than prolonged application—an advantageous property when considering topical formulations. These findings underscore the importance of precise dosing in the therapeutic use of plant-derived compounds and provide a foundation for further investigation into the molecular mechanisms driving the selectivity of these essential oils.

Future studies should aim to identify the specific bioactive compounds responsible for these effects, elucidate their mechanisms of action, and validate these findings in in vivo models. If further confirmed, *L. vera* and *S. fruticosa* could emerge as promising candidates in integrative or adjunctive treatments for skin cancer, potentially enhancing efficacy while reducing the side effects associated with conventional therapies.

The anticancer properties of *J. excelsa*, *L. vera*, and *S. fruticosa* essential oils are not merely due to their cytotoxicity but also involve complex molecular mechanisms that modulate key pathways involved in cancer progression. By targeting these molecular pathways, these oils may help inhibit tumor growth, promote apoptosis, and reduce metastasis, offering a multifaceted approach to cancer treatment [23].

As mentioned earlier, skin cancer, particularly melanoma and non-melanoma skin cancers (NMSCs), is driven by UV-induced oxidative stress, which results in DNA damage and mutagenesis [17,18]. A major molecular mechanism by which the essential oils exert their anticancer effects is through the reduction of oxidative stress. Both Lavandula vera and *S. fruticosa* have demonstrated antioxidant properties by neutralizing free radicals and reducing the formation of reactive oxygen species (ROS). This helps maintain cellular integrity and prevents DNA mutations, which are crucial in the initiation and progression of skin cancer [24,25,26,27].

As another example, α-pinene, found in *J. excelsa*, has been shown to enhance the activity of antioxidant enzymes such as superoxide dismutase (SOD) and catalase, which play pivotal roles in scavenging ROS. By mitigating oxidative damage, these oils not only protect healthy cells but also inhibit the genetic alterations that drive carcinogenesis [28,29].

Another critical mechanism by which essential oils exert their anticancer effects is by modulating the cell cycle and inducing apoptosis in transformed cells. Cancer cells often acquire mutations that allow them to bypass cell cycle checkpoints and resist apoptosis, leading to uncontrolled proliferation.

Both *L. vera* and *S. fruticosa* essential oils influence key regulators of the cell cycle, including cyclins, cyclin-dependent kinases (CDKs), and tumor suppressor proteins such as p53. Studies have shown that linalool (in Lavandula vera) and carnosic acid (in *S. fruticosa*) can induce G0/G1 arrest, preventing malignant cells from progressing through the cell cycle and dividing. This results in reduced cell proliferation in cancerous tissues [24,30,31].

Furthermore, these oils also promote apoptosis through mitochondrial and caspase-dependent pathways. For instance, linalool has been demonstrated to activate caspases-3 and -9, key enzymes that execute the apoptotic process by cleaving vital cellular components. This activation of programmed cell death helps eliminate malignant cells without affecting healthy keratinocytes [30,32].

One of the hallmarks of cancer is its ability to stimulate angiogenesis, the formation of new blood vessels, which supplies nutrients to the growing tumor and facilitates metastasis. Essential oils from *L. vera* and *S. fruticosa* have been found to inhibit angiogenesis by downregulating key pro-angiogenic factors, such as vascular endothelial growth factor (VEGF). By inhibiting VEGF signaling, these oils disrupt the blood supply to tumors, thereby impeding their growth and spread [32].

Moreover, both oils have demonstrated the ability to suppress matrix metalloproteinases (MMPs), enzymes involved in the degradation of the extracellular matrix. This action may reduce the capacity of cancer cells to invade surrounding tissues and metastasize to other organs. These effects are crucial in preventing tumor progression and the spread of skin cancer to distant sites [31,32].

## 5. Conclusions

This study assessed the anticancer effects of essential oils from *Juniperus excelsa* M. Bieb. (Cupressaceae), *Lavandula vera* DC. (Lamiaceae), and *Salvia fruticose* (Mill.) (Lamiaceae) on non-tumorigenic HaCaT, benign A5, and low-grade malignant II4 keratinocytes. All three oils showed significant cytotoxic and antiproliferative effects. While *J. excelsa* was active against both normal and cancerous cells, its lack of selectivity limits its clinical relevance. In contrast, *L. vera* and *S. fruticosa* displayed selective toxicity toward malignant cells, particularly at low concentrations, sparing normal keratinocytes, which suggests their potential for targeted skin cancer therapies. Overall, essential oils from these Mediterranean plants emerge as promising antitumor agents. Future work should identify active compounds and investigate mechanisms such as mitochondrial membrane potential disruption, cytochrome c release, and caspase activation. In vivo studies and clinical trials are needed to evaluate safety, dosing, and possible synergy with standard treatments.

## Figures and Tables

**Table 1:** Different chemical compositions of essential oils.

	Plant Name		*Juniperus excelsa*	*Salvia fruticosa*	*Lavandula vera*
	Yield % (*v*/*w*)		1.17	0.9	1.25
R_i_	Compound ID	Identification			
938	*α*-Pinene	R_i_, MS, CoGC	86.8	0.8	0.1
953	Camphene	R_i_, MS, CoGC	-	-	0.1
980	*β*-Pinene	R_i_, MS, CoGC	2.5	3.2	
993	Myrcene	R_i_, MS, CoGC	3.2	1.4	0.4
1013	*δ*-3-Carene	R_i_, MS	2.4	2.5	
1030	Limonene	R_i_, MS, CoGC	2.2	-	
1034	1,8-Cineole	R_i_, MS, CoGC	-	48.7	2.8
1057	*γ*-Terpinene	R_i_, MS, CoGC	0.3	-	
1074	Linalool oxide	R_i_, MS			0.5
1098	Linalool	R_i_, MS, CoGC			42.5
1105	*α*-Thujone	R_i_, MS	-	0.4	
1138	Menthone	R_i_, MS	-	-	
1145	Camphor	R_i_, MS, CoGC	trace < 0.05	1.1	10.4
1156	Isoborneol				1.1
1166	Borneol				5.6
1168	Lavandulol	R_i_, MS	-	-	0.2
1176	Terpinen-4-ol				10.5
1189	alpha-terpineol	R_i_, MS	0.4	-	0.9
1191	Hexyl butyrate				2.5
1217	Verbenone	R_i_, MS	0.1	-	
1233	Pulegone	R_i_, MS, CoGC	-	-	
1264	Linalyl acetate	8.6			
1289	Lavandulyl acetate				1.9
1329	Piperitone	R_i_, MS, CoGC	-	-	
1343	Piperitenone	R_i_, MS	-	-	
1365	Neryl acetate				0.3
1369	Piperitenone oxide	R_i_, MS			
1383	Geranyl acetate				1.1
1404	(Z)-Caryophyllene				1.1
1415	*β*-Caryophyllene	R_i_, MS, CoGC	-	30.8	
1437	Aromadendrene	R_i_, MS	-	3.3	
1452	*β*-Farnesene				3.1
1455	*α*-Humulene	R_i_, MS	-	2.8	
1463	allo-Aromadendrene	R_i_, MS	-	0.3	
1491	Bicyclogermacrene	R_i_, MS	-	-	
1500	Lavandulyl isovalerate				1.6
1515	*δ*-Cadinene	R_i_, MS	trace < 0.05	0.1	
1566	*α*-Nerolidol				0.2
1577	Caryophyllene oxide				0.5
	Total		97.8	95.5	87.0

Extraction yield (% *v*/*w*) and chemical composition (% relative content) of the essential oils from *Juniperus excelsa*, *Salvia fruticosa*, and *Lavandula vera*. Identification was performed based on Retention Index (Ri), Mass Spectrometry (MS), and Co-injection Gas Chromatography (CoGC).

## Data Availability

The original contributions presented in this study are included in the article. Further inquiries can be directed to the corresponding author(s).
